# Individual notions of fair data sharing from the perspectives of Swiss stakeholders

**DOI:** 10.1186/s12913-021-06906-2

**Published:** 2021-09-23

**Authors:** Lester Darryl Geneviève, Andrea Martani, Bernice Simone Elger, Tenzin Wangmo

**Affiliations:** 1 Institute for Biomedical Ethics, University of Basel, Basel, Switzerland; 2 University Center of Legal Medicine, University of Geneva, Geneva, Switzerland

**Keywords:** Data sharing, Fairness, Health Research

## Abstract

**Background:**

The meaningful sharing of health data between different stakeholders is central to the advancement of science and to improve care offered to individual patients. However, it is important that the interests of individual stakeholders involved in this data sharing ecosystem are taken into account to ensure fair data sharing practices. In this regard, this qualitative study investigates such practices from the perspectives of a subset of relevant Swiss expert stakeholders, using a distributive justice lens.

**Methods:**

Using purposive and snowball sampling methodologies, 48 expert stakeholders from the Swiss healthcare and research domains were recruited for semi-structured interviews. After the experts had consented, the interviews were audio-recorded and transcribed verbatim, but omitting identifying information to ensure confidentiality and anonymity. A thematic analysis using a deductive approach was conducted to identify fair data sharing practices for secondary research purposes. Themes and subthemes were then identified and developed during the analysis.

**Results:**

Three distributive justice themes were identified in the data sharing negotiation processes, and these are: (i) *effort*, which was subcategorized into two subthemes (i.e. a claim to data reciprocity and other reciprocal advantages, and a claim to transparency on data re-use), (ii) *compensation*, which was subcategorized into two subthemes (i.e. a claim to an academic compensation and a claim to a financial compensation), and lastly, (iii) *contribution*, i.e. the significance of data contributions should be matched with a corresponding reward.

**Conclusions:**

This qualitative study provides insights, which could inform policy-making on claims and incentives that encourage Swiss expert stakeholders to share their datasets. Importantly, several claims have been identified and justified under the basis of distributive justice principles, whilst some are more debatable and likely insufficient in justifying data sharing activities. Nonetheless, these claims should be taken seriously and discussed more broadly. Indeed, promoting health research while ensuring that healthcare systems guarantee better services, it is paramount to ensure that solutions developed are sustainable, provide fair criteria for academic careers and promote the sharing of high quality data to advance science.

**Supplementary Information:**

The online version contains supplementary material available at 10.1186/s12913-021-06906-2.

## Background

Over the past few decades, data sharing has become an increasingly discussed topic in the scientific literature. It has further regained impetus following the approval of the European General Data Protection Regulation (GDPR) and its enforcement in 2018 [1]. This growing interest in data sharing also stems from the fact that its potential benefits from both a clinical and research perspective are increasingly underscored. Indeed, it is claimed that data sharing: (i) promotes new scientific discoveries through the re-analysis of shared datasets to test new hypotheses; (ii) helps in building trust and transparency in scientific findings given that their reproducibility can be independently verified; (iii) limits waste in research by preventing duplication of efforts; (iv) accelerates the uptake of research findings into routine clinical practice; and (v) improves the quality of patient care [2–5]. Therefore, proponents of data sharing argue that the latter is justified under two sets of arguments, namely those of an *ethical* and *moral* nature (e.g., reducing safety risks for research participants), and those of a *scientific* and *practical* nature (e.g., ensuring the reproducibility of research findings) [5].

Data sharing can be understood as “any form of release of research [and healthcare] data for use by others” (adapted definition from [6]). In the research context, this can be achieved by either making data available on data repositories (including on the website of researchers and their institutions, or as supplementary material in scientific publications) or by accepting to fulfill requests for data of external researchers [6]. In the United States, the *open data movement* – i.e. making datasets openly available – was encouraged by the 2009 memorandum on “Transparency and Open Government” [7, 8]. It was exemplified by the subsequent launch of *Data. Gov* [7], a US online governmental platform, which is now hosting more than 300,000 freely-available datasets [9]. Recognizing the value of research datasets to advance science beyond their initial contribution, the *open data movement* has also been taken up by the scholarly data publishing ecosystem. This led to data sharing requirements as a pre-publication condition for researchers (e.g., enforced by funders and journals [10]) and the creation of online data repositories for many scientific disciplines. For instance, the *Harvard Dataverse Repository* allows researchers to “open [their] data to the general public, or restrict access and define customizable terms of use” [11].

One important aspect behind the idea of making datasets more open, is to make them re-usable outside the context in which they are initially collected (as demonstrated by the attribution of unique *Digital Object Identifiers* for future citations [11]). In this context, good data management and stewardship are deemed essential components for an effective and appropriate re-use of scholarly datasets. In this regard, the FAIR Data Principles (Findability, Accessibility, Interoperability and Reusability) have been formulated as a guideline to ensure that digital deliverables of scientific research can “become ‘first class citizens’ in the scientific publication ecosystem, where the quality of the publication—and more importantly, the impact of the publication—is a function of its ability to be accurately and appropriately found, reused, and cited over time, by all stakeholders, both human and mechanical” [12].

In spite of the different ways how data can be shared, ultimately the exchange of data between different stakeholders also depends on the willingness of several stakeholders to provide access to their datasets. It is therefore important to understand the disincentives and incentives with respect to data sharing from the perspectives of individual researchers both from a systemic and individual level [13]. In fact, whereas technological solutions (e.g. availability of data repositories) might help, they are unlikely to be the *silver bullet* that would positively influence a researcher’ behavior or attitude with regard to the sharing of data, especially if social or cultural aspects are neglected [6].

There are numerous reasons that undermine or hinder the sharing of health-related data for research purposes. These include among others: (i) ethical and legal barriers, such as the need to safeguard the privacy of data subjects due to the sensitive nature of health-related data; (ii) trust issues, limited expertise and time to carry out successfully data sharing activities, apprehensions regarding potential misinterpretation or misuse of shared datasets; (iii) technical barriers (e.g. differing data standards or limited data linkage capacities), (iv) the lack of systemic attribution mechanisms for shared datasets that would give credit back to the original data collectors, and lastly (v) financial barriers (i.e. preparing a dataset for sharing is a costly procedure) [13–18]. Moreover, barriers may differ based on the individual. Indeed, an early-career researcher is less likely to report data sharing issues linked to limited time availability to deposit a dataset than a middle or late-career researcher [19]. Additionally, openly sharing datasets on online repositories or as supplementary open-access files in scientific publications could expose not only the original data collectors to new threats such as data theft [20], but also research participants to additional privacy risks. Indeed, such open-access datasets – although *anonymized* - might be linked with other publicly available datasets and increase the risk of re-identifying study participants [21].

In cases where data cannot be shared on online repositories, it is possible to negotiate access with the original data collectors. For these cases, it is important to understand which data sharing practices are considered as being fair or unfair, and therefore likely to be adopted or rejected by researchers. In this manuscript, we propose using a distributive justice lens to identify such data sharing practices. These “fair” data sharing practices should not be confused with the FAIR Data Principles described earlier, as further explained in the data analysis part of the methods section.

Hence, this qualitative study explores the individual notions of fair data sharing from the perspectives of a subgroup of relevant Swiss stakeholders through the lenses of distributive justice. We rely on interview data collected through semi-structured interviews conducted with 48 expert stakeholders, in particular those involved predominantly in health services research but also with the inclusion of other experts active in the Swiss healthcare and policy-making fields. We analyzed their views on individual notions of fairness for sharing health data. Through an analytical framework based on the concept of distributive justice, we reflected on those data sharing practices that might be perceived as just or fair and which form part of the negotiation process during which requests to share data by external researchers are considered by data collectors. Hence, this study was conducted to better understand how this negotiation process is taking place between Swiss expert stakeholders in order to tailor recommendations for the Swiss context. In other manuscripts belonging to the same project, the distinct topics of systemic fairness for the sharing of health data and data ownership were treated [13, 22].

## Methods

### Ethics statement

The data for this paper was collected as part of our larger research project titled “advancing SMart solutions and eliminating barriers for the Acquisition, Analysis, and Sharing of Health data in Switzerland” (SMAASH), belonging to the Swiss National Research Programme 74 on Smarter Health Care. The SMAASH project aims to identify barriers and facilitators to the processing of health data in the Swiss context, and it does not fall under the remit of the Swiss Human Research Act (HRA). Therefore, according to Swiss legislation, the SMAASH project is exempt from requiring an ethical approval. This was confirmed by the cantonal ethics committee in Northwest and Central Switzerland (file number: EKNZ req-2017-00810), which stated that general and scientific requirements were both satisfied and that the study did not entail any risk for participants’ health.

### Identification and recruitment of study participants

Study participants had to fulfill the following eligibility criteria for this study: (i) at the time of recruitment, they had to be working in the Swiss healthcare domain (including research), (ii) they were either policymakers or researchers or individuals responsible for the management of health datasets, e.g. disease registries, data linkage institutions, hospital IT infrastructures or any other data initiative (national or regional). They were recruited by purposive – which also included identification of eligible participants via a systematic review [14] that was conducted in an earlier phase of the SMAASH project - and snowball sampling, whereby interviewed participants were asked to recommend additional interviewees for this study. The identified stakeholders were approached via email, and subsequently enrolled for semi-structured interviews after having been informed about, amongst others, the aims of the research project and the data security measures in place to protect their privacy and confidentiality. The recruited 48 expert stakeholders (the majority being in the middle to late stages of their careers) were categorized into three main groups: researchers (*n* = 28), policy-makers (*n* = 10) and lastly, those having a senior position in managing health databases, Information Technology infrastructures or data initiatives (*n* = 10). Study participants provided their verbal consent for the interviews to be audio-recorded after having been provided with consent information and the opportunity to have their additional questions answered by the interviewer. The participants were guaranteed that the resulting recordings would be transcribed verbatim for further analysis, but excluding details that could potentially reveal their identity. Accuracy of the transcripts were also checked by some interviewees who requested it.

### Interview guide and data collection

As this study is part of a larger research project, the interview guide was developed by LDG, AM, BSE and TW to answer the broad research objectives of the SMAASH project. Questions in the interview guide were further enriched and refined via additional information gathered during the simultaneous conduct of the systematic review mentioned in the previous section [14]. The interview guide was then pilot-tested and modified accordingly to ensure that interview questions were clear and easily understandable (annexed). Additional probing questions were also developed for questions judged to require additional or deeper investigation. For instance, concerning the fourth question of the annexed interview guide, probing questions were developed around the interviewee’s general knowledge on the current status of data sharing activities for research in the Swiss context, the challenges encountered in sharing health data and solutions that could be implemented to address them, including general recommendations on how to improve data sharing at the local and international level. Furthermore, other follow-up probes were also asked when needed during the conduct of the interviews.

The data collection period started in May 2018 and ended in September 2019. After receiving training in qualitative interviews, 43 semi-structured interviews (with duration ranging from 38 to 131 min) were conducted by LDG and AM, of which 37 interview sessions were conducted in English, whilst the remaining few (*n* = 6) were conducted either in French, German or Italian depending on the preferences of the study participants. The majority of the interview sessions were one-on-one interviews, with only four being either one-on-two or one-on-three, for a total of 48 expert stakeholders interviewed for this study. The audio recordings were placed in an access-protected folder on a secured server provided by the University of Basel, which was only accessible by members of the research team.

### Data analysis

A thematic analysis of the transcripts was carried out using the qualitative analysis software MAXQDA (versions 18 and 20), and was inspired by Braun and Clarke’s analytical approach [23]. LDG, AM and TW were involved in the preliminary analysis of a sample of the first seven transcripts that led to the development of an initial coding tree and the identification of themes and sub-themes related to the aims of the SMAASH project. In this regard, the SMAASH project aims to identify factors that influence positively or negatively the collection, sharing and linkage of health data. The initial coding tree and the list of themes were then finalized during the ongoing analysis of the remaining transcripts by the authors. The few non-English transcripts were coded and analyzed, and relevant data extracts for this study were translated by one of the authors proficient in the interview language (mother tongue or at least C1 language level).

After the data corpus was entirely coded, the authors met to discuss the main topics stemming from it. Some of the main topics included (i) data ownership issues [22], (ii) Swiss stakeholders’ recommendations to improve the health data infrastructure in the Swiss context [24], (iii) systemic issues hindering the fair sharing of health data [13], and (iv) individual notions of fairness for sharing health data. For the purpose of this study, data extracts pertaining to the individual notions of fairness for sharing health data were gathered by LDG, who subsequently carried out a deductive thematic analysis [23] and identified themes and subthemes that englobed the individual notions of fair sharing of health data through a distributive justice lens.

Distributive justice can be viewed as “the fair distribution of the burdens and benefits of social cooperation among diverse persons with competing needs and claims” [25]. To better identify and evaluate fair data sharing activities between the relevant stakeholders, *desert-based principles* – a set of distributive justice principles – were used as an analytical framework. In this regard, *desert-based principles* can be viewed as either falling under one of these three main classifications: (i) *compensation* (e.g. the original data collectors should receive due rewards for the financial expenses made in collecting, managing and sharing datasets), (ii) *effort* (e.g., the original data collectors should receive due rewards for the efforts they have put in collecting, managing and sharing datasets), and (iii) *contribution* (e.g., the original data collectors should receive due rewards for the significance of their contributions in collecting, managing and sharing of datasets) [26]. Therefore, any data extract that did not include notions of fair or unfair data sharing practices based on the desert-based principles were excluded.

### Research team and reflexivity

At the time of the study, LDG and AM were two doctoral candidates in biomedical ethics, who received training in conducting qualitative interviews in preparation for the field work. AM has a legal background whilst LDG’s background is in medicine and global health. TW, a senior researcher, and BSE, the principal investigator of the SMAASH project, have both extensive qualitative research experience, in particular concerning health research. TW and BSE continuously supervised the analysis part of the study to help limiting misinterpretation and bias due to potential preconceptions held by LDG and AM.

## Results

### Individual notions of fair data sharing

**Fig. 1:**
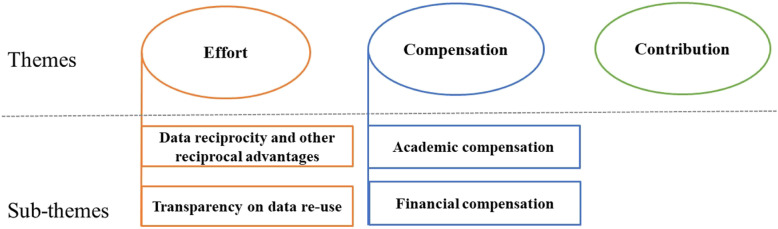
Themes and sub-themes of individual notions of fair data sharing

Three themes pertinent to the desert-based justice principles were identified in the data extracts, namely (i) *effort*, which was further categorized into two sub-themes, (ii) *compensation*, which was also subcategorized into two sub-themes, and (iii) *contribution* (Fig. 1).

### Effort

#### Data reciprocity and other reciprocal advantages

We defined data reciprocity as the actions taken by the original data collector to make data available to the data recipient, only if the former receives in return additional data from the data recipient. Some participants made it clear that their main motivation for sharing data with others resides in the expectation that they shall also receive additional data from the recipients.

“On the personal point of view, I am willing to collaborate with others, and if I share [data], I will also receive” – Res2

Others adopted a more nuanced perception of data reciprocity, highlighting the existence of mutual benefits between the original data collectors and data recipients, but also the need to tailor data sharing requirements to the efforts made by the original data collectors. For instance, one researcher responsible for the management of registries highlighted that given the efforts and resources invested by some of the original data collectors, they did not have to pay to use data from the main database.

“And if people ask for access for data from cohorts, which I lead, [ … ] the (medical specialty) oncologists, they have invested their time and money into collecting data, they get [data] about/of course for free” – Res8

Similarly, another stakeholder stressed the importance to cater for the needs of the original data collectors by providing them in return some valuable information that they could use. The importance of trustful relationships was noted as an important element to ensure the continued sharing of data between the original data collectors and the data recipients.

“We share the data. We make the data useful, also for the data providers, because they can use their statistical data and show something. This contributes to build this trust and I think that it will last a few years and then most of the physicians will provide their data.” – Pol4

Another person involved in health policy-making also noted that, in spite of competing interests (e.g. competition between University Hospitals), University Hospitals have agreed to share data between one another for a common objective: the advancement of the Swiss healthcare system under the framework of a national data initiative.

“I must say they [University hospitals] realize that it is a win-win situation for everyone. And again, I don’t know how much they do really share … if it's 90%, if it's only 10%, if it's 5%, I have no idea. We will see that in the future but at least, I would say those have realized that, they can’t do it alone if they want still do research that's on the high level.” – Pol2

#### Transparency on data re-use

Some study participants discussed transparency with relation to the secondary use and management of the shared datasets in return for their contribution, which was also deemed as an essential component for trustful collaborations. Furthermore, transparency was also considered important to ensure that appropriate data security measures are taken to maintain confidentiality of the shared datasets, and to ascertain clear data ownership and data usage rules.

“But to be in a position to collaborate we need trust. And this is// the first thing we are trying to do is to show transparently what we are doing, what is happening with the data. And to build some sort of a trustful collaboration with the providers of data.” – Pol4

“ … share something if you are aware of what the other partner would do [with] the data or intends to do and how they ensure confidentiality” – Res2

“That's why I think it remains important to really be open and clear and transparent which data belongs to whom and how can it be used.” – Pol8

Because of the duty to protect the patients included in the dataset, one researcher requested that data recipients need to provide detailed and clear information on the duration that the shared datasets will remain in their possession, and that the shared datasets should not be passed further on to any third party.

“[I would share data] With conditions, absolutely. Sure first of all, my first priority is to those patients, I did not inform them in the informed consent. I did not say: “oh I'm going to be passing your data to X, Y, Z you know”. I didn’t feel the need to you know…So it would have to be very clear who am I giving these data to? What are their purposes? How long these data will be available to that person? And you know whether that person can then pass them on further? That I wouldn’t like, I wouldn’t be comfortable with that.” – Res26

Although a lot of researchers requested transparency of future data use in return for their data contribution, this was in practice operationalized by setting up contractual agreements between the original data collectors and the data recipients. For instance, some stakeholders described that the first step in the data sharing process was to initiate a contract with all clauses pertaining to the secondary use of data.

“ … the big step was negotiating a contract. Talking about details, what we are allowed to do, who has access, how are the data stored, where are they stored etc. and things like that. Once we had the contract it was rather simple. They just gave us the data, we knew what we are allowed to do, that was simple” – Res13

“ … we need to sign a contract between the one who is sharing the data and the one who will use it. First to make sure that all the steps of the research project are respected.” – Res9

“If I share data with you, we need to have a contract about what's happen with this data” – Stak4

Indeed, contractual agreements were considered essential for the sharing of health data because they set clear rules on how the shared datasets should be used, and they also provide some protection to the original data collectors. Contractual agreements helped ensure that (i) the shared datasets are less likely to be misused or used illegally (particularly important for potentially identifiable information), (ii) appropriate data security measures had been taken by the data recipients, (iii) publications rights are respected (e.g. avoiding competition for publications or authorship order between original data collectors and recipients), and lastly, (iv) that there is a general acceptation of the rules and conditions of use by both parties involved.

“Well in terms of researchers I would say yes, they have fear that others could publish the data but we address this with the data transfer use agreement. So that has clauses for publication rights, IT-rights and if both parties agree to that then this is solved. On a hospital level actually there is a so-called "Rahmenvertrag" [German for ‘framework agreement’] where all the hospitals agreed to do what they can in order to make data available and share it with each other. So I see the intention is there, that they want to reach this, to come to a data-sharing situation” – Stak7

“That's should be some legal contract between the data sharer and the team which will use it. And of course, there should be some agreement between the two parts on publications, citations… well that's already important.” – Res9

“I must also say I think even if it looks silly but I think it's really good before you share data that you have a contract where it is clear who owns the data, where does it go, who is first author, who is last author … ” – Pol2

Importantly, it was further highlighted that contractual data sharing agreements put into broad daylight the responsibilities that each party has with regard to the shared dataset. Therefore, any breach of the contractual agreement’s rules would be considered irresponsible action.

“With a contract…. there is a reciprocal acceptation of the rules: what happens with the data; what shouldn't or couldn't happen with the data. And then it's a matter of responsibility.” – Pol4

“Again here I would welcome that we have sort of templates what we need to sign to share data. So I still want something in written. You know it's not just via email I send you a link to a dropbox and here you have the data, do whatever you like! [ … ] There is a sort of a responsibility on both sides, those who make data available and those who then take on board data from others. They also have to sign something I think.” – Res17

### Compensation

#### Academic compensation

The sub-theme *academic compensation* refers to the justification of data sharing activities under the conditions that co-authorships in resulting publications or collaborations will be offered in return for the resources and work invested by the original data collectors in collecting and managing datasets. For instance, some researchers described a case where they requested data from a health insurer. In return for the data, the latter requested to be a co-author in subsequent publications, and had the upper hand over the researchers in the negotiation process. One of the researchers found the practice of requesting co-authorship as relatively surprising from a person who is not even competing academically for limited resources (e.g. career and funding opportunities) but could potentially be explained by the insurer’s prior role as an academic researcher. Another interviewee justified the fairness of the health insurer’s actions by referring to the resources the latter invested in the data sharing activities.

“Participant number 3: … there was a power game around the actual data. For example, he said, "I want to be a co-author of the publications." To say, which is surprising because one imagines that he is an administrative [person working for health insurance companies], no? That is, an official. Instead well, if he is an administrative but high-level - he probably also had a doctorate - he would also have had publications [ … ]

Participant number 1: … No, well we do it, but it is a job that takes work [preparing datasets for sharing], it takes time, so we have to have something to return. [speaking of the health insurance point of view]” – Res16a/b/c

Similarly, another researcher demanded to be offered co-authorship in publications that made use of his shared dataset. Interestingly, the researcher also highlighted that the practice of offering co-authorship for shared datasets is more common for certain disciplines. Consequently, the interviewee expressed some frustrations with regard to what is current practice in the discipline of economics, i.e. an acknowledgment for sharing data, which the interviewee deemed as insufficient.

“Participant: The only way you could do that is that you say: "Ok we share the data but we are on each paper that is published with this data" and I think in the medicine that's more common. But in economics if you just collected the data, you end up in an acknowledgement footnote. And you are never (emphasized) a co-author*.*

Interviewer: So your condition for sharing the data would be that you are put as a co-author?

Participant: Yes. That would be one way.” – Res12

#### Financial compensation

This type of compensation refers to the claim that data recipients should cover at least part of the expenses incurred by the original data collectors in the collection, management and/or sharing of datasets. Indeed, financial compensation was often mentioned by our interviewees as a way of receiving recognition for the collection or processing of datasets.

“ … this [data sharing] would have to be done also with some kind of financial compensation” – Res10

Moreover, one researcher highlighted that the monetary value of datasets is gradually being recognized, especially from the perspectives of healthcare professionals. For example, healthcare professionals are now asking for a financial compensation for datasets that they have collected.

“In fact, when we arrived and said, "Give it to us," it didn't seem true. Aside from that they also sold it at a high price. But oh well we had the money and we gave it to him. So - how to say - there is a desire - I say on the part of doctors, therefore health professionals - to produce data and to use them because there is a value, a personal gain.” – Res16a/b/c

Similarly, another expert stakeholder also advocated for a more in-depth discussion to clarify whether there is a need to monetize datasets produced by hospital staff. This was deemed particularly important since the latter often do not receive any benefit for the work done in collecting and managing datasets, which are subsequently used to answer research questions.

“ … people in the hospital are collecting data don't have benefits from this work, other than okay you are a good boy, good data collection from you. I think we are missing something there [ … ] the secondary use of data under/monetization of the secondary use of data is something that need to be clarified.” – Stak8

Going beyond fairness considerations, some researchers also highlighted that financial compensation is necessary not only for the resources invested by the original data collectors in collecting, managing and sharing datasets, but also as a means of ensuring the sustainability of some long-term projects (e.g. in the case of cohort data), and the quality of datasets. One researcher also reported that such practice is not well implemented in Switzerland but seems to be common in other countries.

“And then if that group has money, I think they should pay something towards [data sharing] [ …] if it goes towards a group which can get the grant application and share a little of that for data acquisition. I mean we have done that, I’ve asked for a dataset from a cohort study in England to compare our own results, so we had two cohorts and they will be asked for ten or twenty thousand pounds for instance to own the data. So that is a common use I think in some other countries” – Res8.

Similarly, another researcher made an analogy to data sharing requirements imposed by Swiss Federal institutions (e.g. Federal Statistical Office), whereby researchers need to pay to get access to data. The interviewee highlighted that this is a fair practice that could be implemented by researchers who own datasets.

“You see, when you see that some private companies are selling their data, I already bought them, and even the public one, for example the Federal Office of Statistics is selling some data and in fact, they are selling the work they invested in extraction. So it's quite fair in fact. How will they pay the people doing the work? And it's the same for us. If you want to have very good databases and with data quality management, you have to put some resources on that. So if you want to share the data, it should be funded within the project or funded by the others asking for your data.” – Res9

### Contribution

Contribution here refers to the importance of the original data collectors’ data sharing activities for the attainment of the objectives set by the secondary research project, and that such contribution should be compensated by a matching reward made to the original data collectors. For instance, one researcher explained that original data collectors were made co-authors on a resulting manuscript and authorship order was determined based on the volume of patient data each co-author has contributed.

“Indeed, I also had to put them as authors even if they did not even write an article .... but not even an article. But since they gave me the patients, they became authors. And the order of the authors depended on the number of patients. That is, you gave me a lot, you are first.” – Res16a/b/c

Regarding data sharing between different partners for a specific research project, one researcher highlighted that for data sharing to take place, it is necessary that each partner makes more or less an equivalent data contribution. Otherwise, it could be perceived as prejudicial to those collaborators who have contributed more data, in particular if their larger invested resources and efforts are masked behind the collective endeavor.

« … you have to realize that if you want to share the data, everyone needs to have the data and everyone needs to have more or less the same data. Otherwise, as it is very competitive, you will not necessarily want to share from a personal and scientific point of view what you have made a particular effort for, you have obtained the funds, you have found people, you have made a research project and you don't necessarily want what was a huge job for you to become ... part of a bigger project on the same topic where you lose all personal effort » - Res6

## Discussion

This qualitative study explores the individual notions of fair data sharing from the perspectives of Swiss expert stakeholders, through the lens of distributive justice as an analytical framework. In this regard, this study provides insights on fair or unfair data sharing practices that form part of the negotiation process occurring within the Swiss context, whereby data collectors are requested to provide their data to external researchers. From a distributive justice point of view, individual notions of fairness were justified under the efforts made by the individual researchers in collecting, managing and sharing datasets, and these include (i) a claim to data reciprocity and other reciprocal advantages, and (ii) a claim to transparency regarding data-reuse (which was often operationalized through data transfer contractual agreements). Secondly, individual notions of fairness were also justified under the *compensation* claim, and these include: (i) a claim to an academic compensation in the form of co-authorship on manuscript publications or collaboration opportunities, and (ii) a claim to a financial compensation, that is, the costs of data sharing activities should be at minimum covered by those requesting the data. Lastly, under the *contribution* claim, the significance of the efforts (in terms of data) generated by the original data collectors were mentioned, in particular within multi-partner research collaborations (e.g. inequitable data sharing between the parties involved).

Reciprocity has usually been put forward in national and international ethical frameworks governing data sharing activities [27–29]. In our study, the participants hinted that receiving some reciprocal advantage in return for their contribution was a way to acknowledge their efforts in collecting and managing datasets. Such a view might be related to the competitive Swiss academic environment, where original data collectors might fear not receiving due credit for the efforts put in collecting, managing and sharing their datasets (in terms of unsatisfactory attribution mechanisms or diminishing career opportunities) [13]. Therefore, obtaining additional or equivalent data from recipients represents a motivation to share data without and at the same time losing a competitive advantage as compared to the data receivers.

Another individual motivation put forward by the original data collectors for sharing data was that of having transparency rules on the re-use and storage modalities of the shared datasets by data recipients. Transparency has been extensively discussed in many data sharing frameworks, often in the form of data availability statements for external researchers to confirm or refute the validity and test the reproducibility of certain research findings [30–32]. In contrast, our study shows that there is another dimension to transparency. Indeed, data collectors feel entitled to know all modalities associated with the re-use of their datasets, in the same way as data recipients are entitled to know all modalities associated with the creation of these datasets. Indeed, many interviewees stated that they would request, in exchange for sharing their datasets, that data recipients provide clear information on the intended secondary use, duration of storage, data security measures taken and a reciprocal acceptation of data ownership rules by both parties. These transparency claims are operationalized in practice by legally-binding contractual data transfer/data access agreements, where additional requirements were also stipulated (e.g. publication rights). These agreements are common practice whenever data sharing occurs through a controlled access [30].

Concerning academic compensation, receiving co-authorship opportunities as part of data sharing activities has long been perceived as a fair practice in many scientific fields, especially among researchers. For instance, in an international survey on data sharing perceptions and practices by scientists worldwide, it was observed that almost 60% of scientists (out of 1257) found that it was fair for them to receive co-authorship in exchange of their data, and 61% (out of 1226) found it fair to give co-authorship to the original data collectors if they are using their data. Furthermore, offering collaboration opportunities to the original data collectors was also perceived as fair by the great majority of scientists (81%) in return for using their data [18], a practice also recommended by the *International Committee of Medical Journal Editors* (ICMJE) [33]. These perceptions seem to be also reflected in our qualitative study, whereby co-authorship and collaboration opportunities were mentioned as individual motivations to the sharing of data to compensate for the efforts made by data collectors. However, the question arises whether the simple act of sharing datasets solely justifies co-authorship, as it was suggested by some of our interviewees.

In that regard, the ICMJE also provides a series of four authorship criteria that need to be fulfilled for original data collectors to be given co-authorship in a scientific publication, and refutes the claim that efforts made in the acquisition of data are sufficient on their own to justify co-authorship [34]. One could think of contributorship as another means of receiving credit for the data contribution, in particular if all criteria for authorship are not satisfied. In this regard, Richard Horton once argued that contributorship might help to dismantle inappropriate authorship practices (e.g. scientists receiving undue credit), but it cannot help the scientific community to “find an appropriate, consistent, and reproducible means of judging academic merit” [35]. One promising proposition was made by Bierer, Crosas and Pierce in the form of *data authorship* [36].

It is important to differentiate between “data author” and “data collector”, a term that has been extensively used in this manuscript. The difference between the two resides in the fact that any member of a research team who contributes to the data collection process is considered as an original data collector, but to *qualify* as a data author, one needs to make “substantial contributions to the original acquisition, quality control, and curation of the data, be accountable for all aspects of the accuracy and integrity of the data provided, and ensure that the available data set follows FAIR Guiding Principles” [36]. Moreover, data authorship needs also to be differentiated from manuscript authorship in the sense that data authors are only responsible for the scientific integrity of datasets, but they cannot be held accountable for the content or conclusions of a resulting manuscript, unless they are also listed as its authors. For data authorship to matter, it needs to be well-received and implemented within the scientific community, and recognized by academic institutions, journals, funding and governmental agencies as an additional criterion to reward deserving scientists for their data sharing efforts (e.g. just like the Hirsch-index is currently used in academic evaluations, a “d-index” could be envisaged for data authors) [36]. Moreover, from a distributive justice perspective, data authors could also be offered not only collaboration opportunities, but also opportunities to contribute in a substantial way to future publications in order to obtain co-authorship. This would constitute a fair practice given the efforts and resources they have invested in making the datasets shareable whilst assuming full responsibility for their integrity.

Financial compensation was another discussed aspect, also falling under the perceived individual motivations of data sharing. In our study, financial compensation was perceived as a proper reward to cover part of the costs incurred by the original data collectors in the processing of their respective datasets but also as a means of ensuring the quality and sustainability of the health databases. Our study findings are partly aligned with the scientific literature. For example, in an international survey investigating the perceptions and practices of scientists with regard to data sharing, almost 70% of their participants rejected the idea that it is a fair for data recipients to pay the original data collectors for their datasets [18]. In contrast, Cole and colleagues [37] argued that, in the context of academic medical centers, it is fair to provide financial compensation to cover the costs of data acquisition, but stressed that it is crucial that data sharing is not being promoted for financial gains. Indeed, if financial compensation for data sharing will be widely implemented in academia, it is important that the negotiation process between the original data collectors and data recipients to be fair and transparent, with the primary objective of recovering the costs of data processing incurred by data collectors. Therefore, there should be some clear and consistent criteria on how to calculate the minimum fee that data recipients would have to pay to access data, so that the latter are not disadvantaged during the negotiation process with the downside effect of hindering data sharing. In this regard, one could learn from the data access cost calculation methods employed by NHS (National Health Service) Digital, who provide data not only to researchers but also to clinicians and commissioners for the improvement of NHS services [38].

Under the contribution principle, mechanisms to compensate for the quantity and importance of data contributions made by the original data collectors were rarely discussed. Some of our interviewees explained that in some cases, the original data collectors explicitly stated that their degree of contribution to data sharing activities needed to be matched with a corresponding reward (e.g. in the form of authorship order in publications derived from the shared datasets). Others stated that they will be reluctant to engage in data sharing activities if all research collaborators did not contribute an equivalent amount of data. In contrast to certain academic disciplines where authorship order has no value (e.g. in economics or mathematics where authors are listed in alphabetical order), in health research and other disciplines authorship order plays a central role in defining the specific contributions made by each author and also for the academic reward mechanism - in particular how promotion in academia is often linked to the number of first- or last-author publications [39].

Therefore, in health research, authorship order aligns with distributive justice principles, since each author’s contribution “should be assigned a proportionate and fair share of the overall value of the publication” [39]. Nonetheless, defining precisely the contribution of each author to the value of a manuscript is a challenging task and consequently, authorship order based on the amount of data contributed by each research collaborator offers a weak assessment of their academic merit [39]. Additionally, irrespective of their data contributions, all authors need to take full responsibility for the entire content of the manuscript whilst guaranteeing its scientific integrity (see ICMJE criteria for authorship [34]), which further undermines the claim that authorship order is dependent on the amount of data contributed by each author.

### Study limitations

This qualitative study has some limitations. Firstly, the findings of this study might be biased by the seniority of our study participants. Indeed, the majority of our participants were either late- or middle-career stakeholders, and therefore individual notions related to fair data sharing from the perspectives of early-career participants might have been underrepresented or neglected. Secondly, our interview sessions had to be adapted to the needs and expectations of our expert stakeholders, some of whom preferred to have one-on-two or one-on-three interviews rather than the common one-on-one interview. By their nature, group interviews are more subject to group dynamics, which could have influenced the reporting of elements deemed more important for the group of interviewees rather than for the individual. Thirdly, the development of our interview guide was informed by issues related to data sharing that are more predominant in the Swiss context (e.g. those identified in our systematic review [14]). Fourthly, we also acknowledge that this study offers only an initial assessment of the issues identified in the Swiss context (some being blurry concepts) and needed further investigation. Therefore, these issues were explored, clarified and discussed with relevant expert stakeholders during a follow-up Delphi-based workshop [40]. Finally, like every other qualitative study, our findings are not generalizable and may be affected by social desirability bias, where participants may have tended to discuss expected ethical concerns.

## Conclusions

This qualitative study provides insights that could inform policy-making on individual motivations that need to be accounted for to promote fair data sharing from the perspectives of Swiss expert stakeholders. These include claims to data reciprocity and other reciprocal advantages, co-authorship and collaboration opportunities, transparency on data-reuse, financial compensation, and a contribution claim based on the significance of the data contributions made by the original data collectors. While the appropriateness of some of these claims may be debatable, they should be taken seriously and discussed more openly. In order to promote health research whilst improving the quality of the health systems, these individual notions of fair data sharing deserve to be considered when promoting data sharing activities, paying particular attention to sustainable solutions that provide fair criteria for academic careers and increase high data quality and accessibility to advance science.

## Supplementary Information

**Additional file 1.** Interview guide.

## Data Availability

The dataset supporting the findings of this study is available from the corresponding author on reasonable request. The interview guide is annexed.

## Abbreviations

EHR: Electronic Health Record
GDPR: General Data Protection Regulation
ICMJE: International Committee of Medical Journal Editors
NHS: National Health Service
SMAASH: Advancing SMart solutions and eliminating barriers for the Acquisition, Analysis, and Sharing of Health data in Switzerland
